# 3-(2-Bromo­phenyl­sulfin­yl)-2,5,7-trimethyl-1-benzo­furan

**DOI:** 10.1107/S1600536813019867

**Published:** 2013-07-24

**Authors:** Hong Dae Choi, Pil Ja Seo, Uk Lee

**Affiliations:** a Department of Chemistry, Dongeui University, San 24 Kaya-dong, Busanjin-gu, Busan 614-714, Republic of Korea; b Department of Chemistry, Pukyong National University, 599-1 Daeyeon 3-dong, Nam-gu, Busan 608-737, Republic of Korea

## Abstract

In the title compound, C_17_H_15_BrO_2_S, both the benzo­furan and 2-bromo­phenyl rings are virtually planar, with r.m.s. deviations of 0.009 (2) and 0.006 (2) Å, respectively. The dihedral angle between these mean planes is 89.31 (7)°. In the crystal, mol­ecules are linked *via* pairs of C—H⋯π inter­actions into inversion dimers. These dimers are further linked by C—H⋯π inter­actions into supra­molecular chains running along the *b* axis. In addition, C—S⋯π inter­actions, with an S-to-ring-centroid distance of 3.50 (2) Å, are observed between inversion-related dimers.

## Related literature

For background information and the crystal structures of related compounds, see: Choi *et al.* (2010 ▶, 2012 ▶).
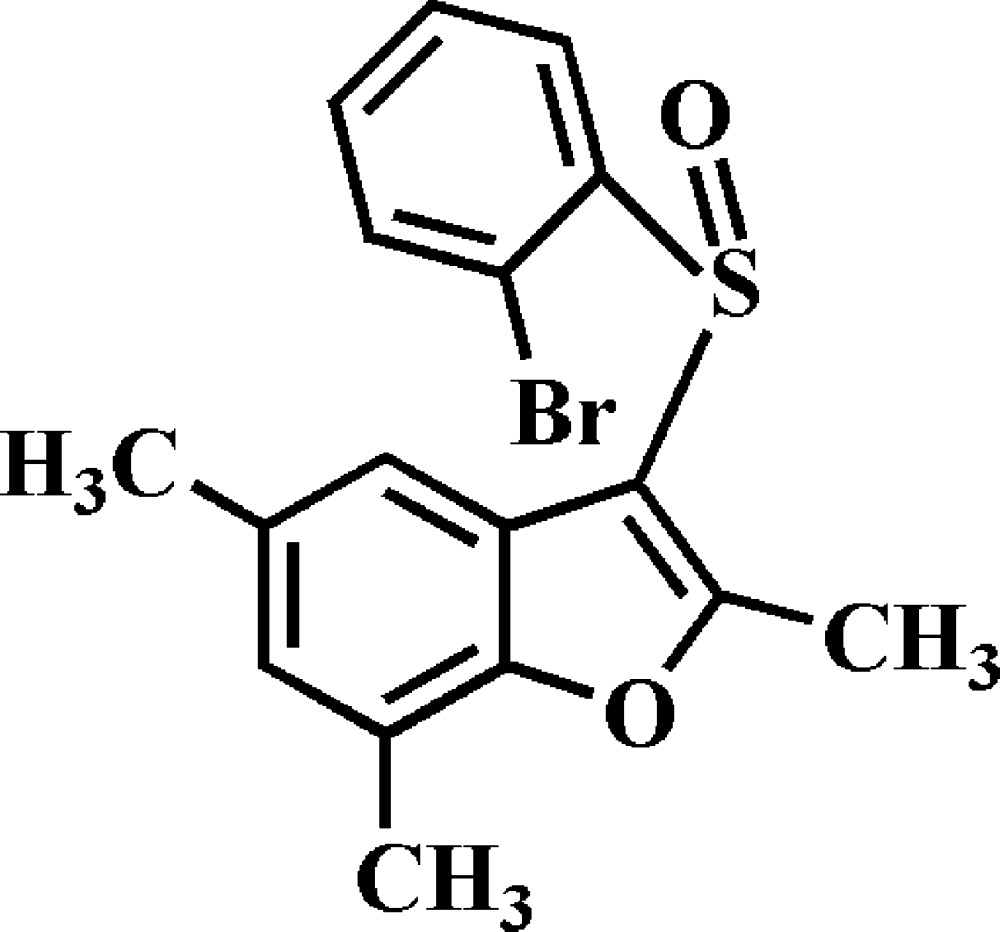


## Experimental

### 

#### Crystal data

C_17_H_15_BrO_2_S
*M* *_r_* = 363.26
Triclinic, 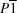

*a* = 7.540 (3) Å
*b* = 8.415 (3) Å
*c* = 12.722 (4) Å
α = 98.933 (19)°
β = 105.001 (19)°
γ = 93.530 (18)°
*V* = 765.9 (5) Å^3^
*Z* = 2
Mo *K*α radiation
μ = 2.82 mm^−1^
*T* = 173 K
0.32 × 0.26 × 0.20 mm

#### Data collection

Bruker SMART APEXII CCD diffractometer
Absorption correction: multi-scan (*SADABS*; Bruker, 2009 ▶) *T*_min_ = 0.522, *T*_max_ = 0.746
12648 measured reflections
3304 independent reflections
2857 reflections with *I* > 2σ(*I*)
*R*_int_ = 0.038

#### Refinement

*R*[*F* ^2^ > 2σ(*F* ^2^)] = 0.033
*wR*(*F* ^2^) = 0.085
*S* = 1.12
3304 reflections
193 parameters
H-atom parameters constrained
Δρ_max_ = 0.29 e Å^−3^
Δρ_min_ = −0.54 e Å^−3^

### 

Data collection: *APEX2* (Bruker, 2009 ▶); cell refinement: *SAINT* (Bruker, 2009 ▶); data reduction: *SAINT*; program(s) used to solve structure: *SHELXS97* (Sheldrick, 2008 ▶); program(s) used to refine structure: *SHELXL97* (Sheldrick, 2008 ▶); molecular graphics: *ORTEP-3 for Windows* (Farrugia, 2012 ▶) and *DIAMOND* (Brandenburg, 1998 ▶); software used to prepare material for publication: *SHELXL97*.

## Supplementary Material

Crystal structure: contains datablock(s) global, I. DOI: 10.1107/S1600536813019867/fj2637sup1.cif

Structure factors: contains datablock(s) I. DOI: 10.1107/S1600536813019867/fj2637Isup2.hkl

Supplementary material file. DOI: 10.1107/S1600536813019867/fj2637Isup3.cml

Additional supplementary materials:  crystallographic information; 3D view; checkCIF report

## Figures and Tables

**Table 1:** Hydrogen-bond geometry (Å, °) *Cg*1 and *Cg*2 are the centroids of the C2–C7 benzene ring and the C1/C2/C7/O1/C8 furan ring, respectively.

*D*—H⋯*A*	*D*—H	H⋯*A*	*D*⋯*A*	*D*—H⋯*A*
C9—H9*C*⋯*Cg*1^i^	0.98	2.92	3.594 (3)	127
C16—H16⋯*Cg*2^ii^	0.95	2.78	3.551 (3)	139

Symmetry codes: (i) 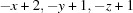; (ii) 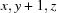.

**Fig. 1.**
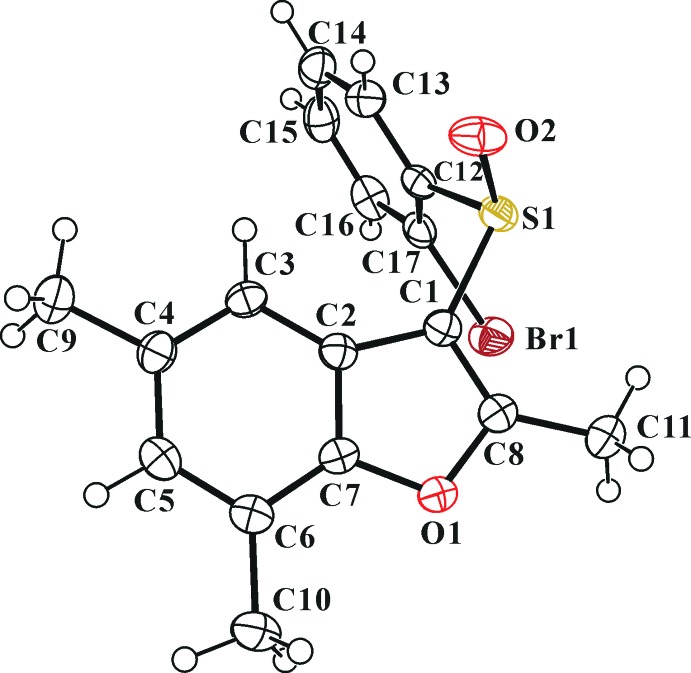
The molecular structure of the title compound with the atom numbering scheme. Displacement ellipsoids are drawn at the 50% probability level. H atoms are presented as small spheres of arbitrary radius.

**Fig. 2.**
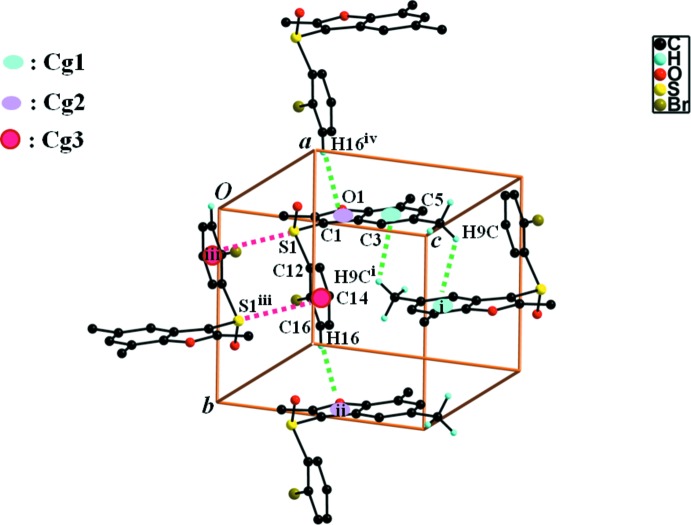
A view of the C–H···π and C–S···π interactions (dotted lines) in the crystal structure of the title compound. H atoms non-participating in hydrogen-bonding were omitted for clarity. [Symmetry codes: (i) - x + 2, - y + 1, - z + 1; (ii) x, y + 1, z; (iii) - x + 1, - y + 1, - z; (iv) x, y - 1, z.]

## supplementary crystallographic information

### Comment

As a part of our oning study of 2,5,7-trimethyl-1-benzofuran derivatives containing 4-fluorophenylsulfinyl (Choi *et al.*, 2010) and 4-bromophenylsulfinyl (Choi *et al.*, 2012) substituents in 3-postion, we report herein the crystal structure of the title compound.

In the title molecule (Fig. 1), the benzofuran unit is essentially planar, with a mean deviation of 0.009 (2) Å from the least-squares plane defined by the nine constituent atoms. The 2-bromophenyl ring is essentially planar, with a mean deviation of 0.006 (2) Å from the least-squares plane defined by the six constituent atoms. The dihedral angle formed by the mean plane of the benzofuran ring system and the mean plane of the 2-bromophenyl ring is 89.31 (7)°. In the crystal packing (Fig. 2), molecules are linked *via* pairs of C–H···π interactions (Table 1, Cg1 is the centroid of the C2-C7 benzene ring) into inversion dimers. These dimers are further linked by C–H···π interactions (Table 1, Cg2 is the centroid of C1/C2/C7/O1/C8 furan ring) into supramolecular chains running along the *b-axis* direction. In addition, there are weak intermolecular S···π interactions between the sulfur atom and the centroid of the 2-bromophenyl ring (C12-C17) of an adjacent molecule, with a S1···Cg3^iii^ being 3.508 (2) Å, resulting in inversion-related dimers.

### Experimental

3-Chloroperoxybenzoic acid (77%, 224 mg, 1.0 mmol) was added in small portions to a stirred solution of 3-(2-bromophenylsulfanyl)-2,5,7-trimethyl-1-benzofuran (312 mg, 0.9 mmol) in dichloromethane (40 mL) at 273 K. After being stirred at room temperature for 4h, the mixture was washed with saturated sodium bicarbonate solution and the organic layer was separated, dried over magnesium sulfate, filtered and concentrated at reduced pressure. The residue was purified by column chromatography (hexane-ethyl acetate, 2:1 v/v) to afford the title compound as a colorless solid [yield 68%, m.p. 457-458 K; *R*_f_ = 0.53 (hexane-ethyl acetate, 2:1 v/v)]. Single crystals suitable for X-ray diffraction were prepared by slow evaporation of a solution of the title compound in ethyl acetate at room temperature.

### Refinement

All H atoms were positioned geometrically and refined using a riding model, with C–H = 0.95 Å for aryl, 0.99 Å for methyl H atoms, respectively. *U*_iso_(H) = 1.2*U*_eq_(C) for aryl and 1.5*U*_eq_(C) for methyl H atoms. The positions of methyl hydrogens were optimized rotationally.

### Crystal data

**Table:**

C_17_H_15_BrO_2_S	*Z* = 2
*M**_r_* = 363.26	*F*(000) = 368
Triclinic, *P*1	*D*_x_ = 1.575 Mg m^−^^3^
Hall symbol: -P 1	Melting point = 457–458 K
*a* = 7.540 (3) Å	Mo *K*α radiation, λ = 0.71073 Å
*b* = 8.415 (3) Å	Cell parameters from 6758 reflections
*c* = 12.722 (4) Å	θ = 2.7–28.3°
α = 98.933 (19)°	µ = 2.82 mm^−^^1^
β = 105.001 (19)°	*T* = 173 K
γ = 93.530 (18)°	Block, colourless
*V* = 765.9 (5) Å^3^	0.32 × 0.26 × 0.20 mm

### Data collection

**Table:**

Bruker SMART APEXII CCD diffractometer	3304 independent reflections
Radiation source: rotating anode	2857 reflections with *I* > 2σ(*I*)
Graphite multilayer monochromator	*R*_int_ = 0.038
Detector resolution: 10.0 pixels mm^-1^	θ_max_ = 27.0°, θ_min_ = 2.5°
φ and ω scans	*h* = −9→9
Absorption correction: multi-scan (*SADABS*; Bruker, 2009)	*k* = −9→10
*T*_min_ = 0.522, *T*_max_ = 0.746	*l* = −16→16
12648 measured reflections	

### Refinement

**Table:**

Refinement on *F*^2^	Primary atom site location: structure-invariant direct methods
Least-squares matrix: full	Secondary atom site location: difference Fourier map
*R*[*F*^2^ > 2σ(*F*^2^)] = 0.033	Hydrogen site location: difference Fourier map
*wR*(*F*^2^) = 0.085	H-atom parameters constrained
*S* = 1.12	*w* = 1/[σ^2^(*F*_o_^2^) + (0.0362*P*)^2^ + 0.4612*P*] where *P* = (*F*_o_^2^ + 2*F*_c_^2^)/3
3304 reflections	(Δ/σ)_max_ = 0.001
193 parameters	Δρ_max_ = 0.29 e Å^−^^3^
0 restraints	Δρ_min_ = −0.54 e Å^−^^3^

### Special details

**Table:**

Geometry. All esds (except the esd in the dihedral angle between two l.s. planes) are estimated using the full covariance matrix. The cell esds are taken into account individually in the estimation of esds in distances, angles and torsion angles; correlations between esds in cell parameters are only used when they are defined by crystal symmetry. An approximate (isotropic) treatment of cell esds is used for estimating esds involving l.s. planes.
Refinement. Refinement of F^2^ against ALL reflections. The weighted R-factor wR and goodness of fit S are based on F^2^, conventional R-factors R are based on F, with F set to zero for negative F^2^. The threshold expression of F^2^ > 2sigma(F^2^) is used only for calculating R-factors(gt) etc. and is not relevant to the choice of reflections for refinement. R-factors based on F^2^ are statistically about twice as large as those based on F, and R- factors based on ALL data will be even larger.

### Fractional atomic coordinates and isotropic or equivalent isotropic displacement parameters (Å^2^)

**Table:**

	*x*	*y*	*z*	*U*_iso_*/*U*_eq_	
Br1	0.37567 (4)	0.55880 (4)	0.20521 (2)	0.03750 (11)	
S1	0.60544 (9)	0.28866 (8)	0.08301 (5)	0.02716 (15)	
O1	0.5429 (2)	0.1218 (2)	0.34388 (13)	0.0266 (4)	
O2	0.7458 (3)	0.2104 (2)	0.03489 (15)	0.0389 (5)	
C1	0.6293 (3)	0.2361 (3)	0.21447 (19)	0.0240 (5)	
C2	0.7898 (3)	0.2554 (3)	0.30929 (19)	0.0241 (5)	
C3	0.9721 (3)	0.3263 (3)	0.3373 (2)	0.0261 (5)	
H3	1.0173	0.3768	0.2862	0.031*	
C4	1.0854 (3)	0.3215 (3)	0.4414 (2)	0.0281 (5)	
C5	1.0155 (4)	0.2476 (3)	0.5162 (2)	0.0310 (6)	
H5	1.0956	0.2466	0.5872	0.037*	
C6	0.8355 (4)	0.1761 (3)	0.4916 (2)	0.0291 (5)	
C7	0.7280 (3)	0.1826 (3)	0.3862 (2)	0.0249 (5)	
C8	0.4873 (3)	0.1568 (3)	0.2387 (2)	0.0258 (5)	
C9	1.2833 (4)	0.3978 (4)	0.4766 (2)	0.0370 (6)	
H9A	1.3071	0.4537	0.4187	0.056*	
H9B	1.3667	0.3135	0.4883	0.056*	
H9C	1.3043	0.4757	0.5455	0.056*	
C10	0.7603 (4)	0.0990 (4)	0.5728 (2)	0.0387 (7)	
H10A	0.6570	0.1553	0.5876	0.058*	
H10B	0.8578	0.1065	0.6419	0.058*	
H10C	0.7173	−0.0151	0.5416	0.058*	
C11	0.2921 (3)	0.1021 (4)	0.1772 (2)	0.0347 (6)	
H11A	0.2629	0.1425	0.1071	0.052*	
H11B	0.2110	0.1442	0.2215	0.052*	
H11C	0.2734	−0.0164	0.1622	0.052*	
C12	0.6930 (3)	0.5004 (3)	0.12476 (18)	0.0244 (5)	
C13	0.8517 (3)	0.5498 (3)	0.0974 (2)	0.0290 (5)	
H13	0.9179	0.4712	0.0665	0.035*	
C14	0.9149 (4)	0.7125 (3)	0.1146 (2)	0.0350 (6)	
H14	1.0229	0.7453	0.0947	0.042*	
C15	0.8198 (4)	0.8273 (3)	0.1610 (2)	0.0364 (6)	
H15	0.8627	0.9389	0.1728	0.044*	
C16	0.6626 (4)	0.7803 (3)	0.1903 (2)	0.0338 (6)	
H16	0.5992	0.8590	0.2235	0.041*	
C17	0.5983 (3)	0.6167 (3)	0.17078 (19)	0.0267 (5)	

### Atomic displacement parameters (Å^2^)

**Table:**

	*U*^11^	*U*^22^	*U*^33^	*U*^12^	*U*^13^	*U*^23^
Br1	0.03110 (16)	0.04774 (19)	0.04011 (17)	0.01447 (12)	0.01611 (12)	0.01220 (13)
S1	0.0323 (3)	0.0273 (3)	0.0219 (3)	0.0011 (3)	0.0082 (3)	0.0035 (2)
O1	0.0258 (8)	0.0286 (9)	0.0269 (9)	0.0010 (7)	0.0092 (7)	0.0072 (7)
O2	0.0568 (12)	0.0300 (10)	0.0380 (10)	0.0081 (9)	0.0281 (9)	0.0033 (8)
C1	0.0269 (12)	0.0210 (12)	0.0247 (12)	0.0027 (9)	0.0074 (10)	0.0046 (9)
C2	0.0271 (12)	0.0218 (12)	0.0234 (12)	0.0027 (10)	0.0079 (10)	0.0024 (9)
C3	0.0280 (12)	0.0229 (12)	0.0298 (13)	0.0013 (10)	0.0106 (10)	0.0074 (10)
C4	0.0260 (12)	0.0253 (12)	0.0320 (13)	0.0019 (10)	0.0070 (10)	0.0038 (10)
C5	0.0332 (13)	0.0327 (14)	0.0263 (13)	0.0063 (11)	0.0049 (11)	0.0068 (11)
C6	0.0330 (13)	0.0290 (13)	0.0273 (13)	0.0053 (11)	0.0101 (11)	0.0073 (10)
C7	0.0254 (12)	0.0240 (12)	0.0273 (12)	0.0027 (10)	0.0103 (10)	0.0052 (10)
C8	0.0269 (12)	0.0233 (12)	0.0277 (12)	0.0033 (10)	0.0092 (10)	0.0030 (10)
C9	0.0273 (13)	0.0411 (16)	0.0398 (15)	0.0011 (12)	0.0051 (12)	0.0061 (12)
C10	0.0423 (16)	0.0457 (17)	0.0326 (15)	0.0035 (13)	0.0128 (13)	0.0163 (13)
C11	0.0268 (13)	0.0398 (16)	0.0361 (15)	−0.0018 (12)	0.0071 (11)	0.0072 (12)
C12	0.0269 (12)	0.0274 (12)	0.0191 (11)	0.0046 (10)	0.0039 (9)	0.0078 (9)
C13	0.0296 (13)	0.0300 (13)	0.0296 (13)	0.0062 (11)	0.0099 (11)	0.0077 (10)
C14	0.0291 (13)	0.0376 (15)	0.0368 (15)	0.0016 (12)	0.0046 (11)	0.0099 (12)
C15	0.0379 (15)	0.0276 (14)	0.0371 (15)	−0.0001 (12)	−0.0009 (12)	0.0060 (11)
C16	0.0391 (15)	0.0277 (14)	0.0305 (14)	0.0106 (11)	0.0027 (11)	0.0018 (11)
C17	0.0245 (12)	0.0334 (14)	0.0212 (11)	0.0076 (10)	0.0027 (10)	0.0064 (10)

### Geometric parameters (Å, º)

**Table:**

Br1—C17	1.896 (3)	C9—H9A	0.9800
S1—O2	1.4913 (19)	C9—H9B	0.9800
S1—C1	1.763 (2)	C9—H9C	0.9800
S1—C12	1.809 (3)	C10—H10A	0.9800
O1—C8	1.378 (3)	C10—H10B	0.9800
O1—C7	1.392 (3)	C10—H10C	0.9800
C1—C8	1.354 (3)	C11—H11A	0.9800
C1—C2	1.451 (3)	C11—H11B	0.9800
C2—C7	1.392 (3)	C11—H11C	0.9800
C2—C3	1.397 (3)	C12—C13	1.388 (3)
C3—C4	1.386 (4)	C12—C17	1.391 (3)
C3—H3	0.9500	C13—C14	1.387 (4)
C4—C5	1.405 (4)	C13—H13	0.9500
C4—C9	1.512 (4)	C14—C15	1.387 (4)
C5—C6	1.387 (4)	C14—H14	0.9500
C5—H5	0.9500	C15—C16	1.384 (4)
C6—C7	1.387 (4)	C15—H15	0.9500
C6—C10	1.509 (4)	C16—C17	1.393 (4)
C8—C11	1.483 (3)	C16—H16	0.9500
			
O2—S1—C1	107.88 (11)	H9A—C9—H9C	109.5
O2—S1—C12	105.17 (11)	H9B—C9—H9C	109.5
C1—S1—C12	99.38 (11)	C6—C10—H10A	109.5
C8—O1—C7	106.49 (18)	C6—C10—H10B	109.5
C8—C1—C2	108.3 (2)	H10A—C10—H10B	109.5
C8—C1—S1	121.31 (19)	C6—C10—H10C	109.5
C2—C1—S1	130.28 (18)	H10A—C10—H10C	109.5
C7—C2—C3	119.3 (2)	H10B—C10—H10C	109.5
C7—C2—C1	104.2 (2)	C8—C11—H11A	109.5
C3—C2—C1	136.5 (2)	C8—C11—H11B	109.5
C4—C3—C2	118.5 (2)	H11A—C11—H11B	109.5
C4—C3—H3	120.8	C8—C11—H11C	109.5
C2—C3—H3	120.8	H11A—C11—H11C	109.5
C3—C4—C5	119.9 (2)	H11B—C11—H11C	109.5
C3—C4—C9	120.8 (2)	C13—C12—C17	119.0 (2)
C5—C4—C9	119.3 (2)	C13—C12—S1	117.37 (18)
C6—C5—C4	123.4 (2)	C17—C12—S1	123.18 (19)
C6—C5—H5	118.3	C14—C13—C12	120.8 (2)
C4—C5—H5	118.3	C14—C13—H13	119.6
C7—C6—C5	114.6 (2)	C12—C13—H13	119.6
C7—C6—C10	122.2 (2)	C13—C14—C15	119.7 (3)
C5—C6—C10	123.2 (2)	C13—C14—H14	120.2
C6—C7—C2	124.4 (2)	C15—C14—H14	120.2
C6—C7—O1	124.9 (2)	C16—C15—C14	120.4 (3)
C2—C7—O1	110.7 (2)	C16—C15—H15	119.8
C1—C8—O1	110.3 (2)	C14—C15—H15	119.8
C1—C8—C11	133.8 (2)	C15—C16—C17	119.5 (2)
O1—C8—C11	115.9 (2)	C15—C16—H16	120.2
C4—C9—H9A	109.5	C17—C16—H16	120.2
C4—C9—H9B	109.5	C12—C17—C16	120.6 (2)
H9A—C9—H9B	109.5	C12—C17—Br1	121.3 (2)
C4—C9—H9C	109.5	C16—C17—Br1	118.05 (19)
			
O2—S1—C1—C8	−121.0 (2)	C8—O1—C7—C6	178.3 (2)
C12—S1—C1—C8	129.6 (2)	C8—O1—C7—C2	0.0 (2)
O2—S1—C1—C2	54.9 (3)	C2—C1—C8—O1	0.0 (3)
C12—S1—C1—C2	−54.5 (2)	S1—C1—C8—O1	176.70 (16)
C8—C1—C2—C7	0.0 (3)	C2—C1—C8—C11	−179.9 (3)
S1—C1—C2—C7	−176.30 (19)	S1—C1—C8—C11	−3.2 (4)
C8—C1—C2—C3	−178.6 (3)	C7—O1—C8—C1	0.0 (3)
S1—C1—C2—C3	5.1 (4)	C7—O1—C8—C11	179.9 (2)
C7—C2—C3—C4	0.1 (3)	O2—S1—C12—C13	5.5 (2)
C1—C2—C3—C4	178.5 (3)	C1—S1—C12—C13	117.0 (2)
C2—C3—C4—C5	−0.6 (4)	O2—S1—C12—C17	177.91 (19)
C2—C3—C4—C9	−179.4 (2)	C1—S1—C12—C17	−70.5 (2)
C3—C4—C5—C6	0.5 (4)	C17—C12—C13—C14	−0.5 (4)
C9—C4—C5—C6	179.3 (2)	S1—C12—C13—C14	172.26 (19)
C4—C5—C6—C7	0.1 (4)	C12—C13—C14—C15	0.8 (4)
C4—C5—C6—C10	−179.2 (3)	C13—C14—C15—C16	0.1 (4)
C5—C6—C7—C2	−0.7 (4)	C14—C15—C16—C17	−1.3 (4)
C10—C6—C7—C2	178.6 (2)	C13—C12—C17—C16	−0.7 (3)
C5—C6—C7—O1	−178.7 (2)	S1—C12—C17—C16	−173.02 (19)
C10—C6—C7—O1	0.6 (4)	C13—C12—C17—Br1	177.67 (18)
C3—C2—C7—C6	0.6 (4)	S1—C12—C17—Br1	5.3 (3)
C1—C2—C7—C6	−178.3 (2)	C15—C16—C17—C12	1.6 (4)
C3—C2—C7—O1	178.9 (2)	C15—C16—C17—Br1	−176.83 (19)
C1—C2—C7—O1	0.0 (3)		

### Hydrogen-bond geometry (Å, º)

Cg1 and Cg2 are the centroids of the C2-C7 benzene ring and the C1/C2/C7/O1/C8 furan ring, respectively.

**Table:**

*D*—H···*A*	*D*—H	H···*A*	*D*···*A*	*D*—H···*A*
C9—H9*C*···*Cg*1^i^	0.98	2.92	3.594 (3)	127
C16—H16···*Cg*2^ii^	0.95	2.78	3.551 (3)	139

Symmetry codes: (i) −*x*+2, −*y*+1, −*z*+1; (ii) *x*, *y*+1, *z*.
